# Brain Signals of Face Processing as Revealed by Event-Related Potentials

**DOI:** 10.1155/2015/514361

**Published:** 2015-06-16

**Authors:** Ela I. Olivares, Jaime Iglesias, Cristina Saavedra, Nelson J. Trujillo-Barreto, Mitchell Valdés-Sosa

**Affiliations:** ^1^Departamento de Psicología Biológica y de la Salud, Facultad de Psicología, Universidad Autónoma de Madrid, 28049 Madrid, Spain; ^2^División de Psicología, Colegio Universitario Cardenal Cisneros, 28006 Madrid, Spain; ^3^Institute of Brain, Behaviour and Mental Health, Centre for Clinical and Cognitive Neuroscience, University of Manchester, Manchester M13 9PL, UK; ^4^Centro de Neurociencias de Cuba, 11600 Havana, Cuba

## Abstract

We analyze the functional significance of different event-related potentials (ERPs) as electrophysiological indices of face perception and face recognition, according to cognitive and neurofunctional models of face processing. Initially, the processing of faces seems to be supported by early extrastriate occipital cortices and revealed by modulations of the occipital P1. This early response is thought to reflect the detection of certain primary structural aspects indicating the presence *grosso modo* of a face within the visual field. The posterior-temporal N170 is more sensitive to the detection of faces as complex-structured stimuli and, therefore, to the presence of its distinctive organizational characteristics prior to within-category identification. In turn, the relatively late and probably more rostrally generated N250r and N400-like responses might respectively indicate processes of access and retrieval of face-related information, which is stored in long-term memory (LTM). New methods of analysis of electrophysiological and neuroanatomical data, namely, dynamic causal modeling, single-trial and time-frequency analyses, are highly recommended to advance in the knowledge of those brain mechanisms concerning face processing.

## 1. Objective

The present work is intended to offer a comprehensive review of the literature regarding those evoked brain responses related to face perception and face recognition. Moreover, we stress the pertinence of using new approaches to better understand the functional meaning of such responses and the underlying neural mechanisms. Firstly, we analyze the theoretical framework (inspired by cognitive psychology, neuropsychology, and, more recently, neuroimaging studies) that has been most frequently used to interpret ERP studies of face processing. We then dedicate a section to each of the clusters of ERP components that have been related to different stages of face processing and examine their possible relationship with the posited nodes of the face processing network derived from neuroimaging studies. In the next section, we consider recent findings derived from new methodological approaches such as dynamic causal modeling, single trial and time-frequency analyses. Finally, the conclusions are set out.

This review will be limited to studies of face structural processing which eventually leads to face recognition (i.e., recognizing a person by seeing her/his face). Other aspects of face processing (recognition of emotional expressions, gaze direction, lip reading, and so on) merit special attention and are beyond the scope of the article.

## 2. Theoretical Framework on Face Processing

### 2.1. Cognitive and Neurofunctional Models Derived from Functional Magnetic Resonance (fMRI) Studies

Conceptualizations on cognitive operations underlying face recognition have been largely influenced by the seminal model of Bruce and Young [1]. This model assumes that face recognition is achieved, after an initial stage of visual analysis, by sequential access to visual-structural and verbal-semantic codes in long-term memory (LTM). The structural codes concerning the physical appearance of each known individual face, with information about the shape of facial features (lips, eyes, etc.) as well as their spatial configuration, are contained in Face Recognition Units (“FRUs”). These memory units are assumed to be specific to the face domain. The verbal-semantic codes comprise personal biographical information (occupation, context where an individual is usually seen, etc.) contained in Person Identity Nodes (PINs) that are in turn connected to verbal codes for the corresponding name. Subsequent interactive activation implementations of Bruce and Young's model have provided working models to demonstrate, through simulation, certain empirical phenomena like semantic priming, repetition priming, cross-modal cueing, and distinctiveness in face recognition [2, 3].

A basic assumption of Bruce and Young's model is that FRUs, PINs, and name codes are activated in a strictly sequential mode. However, the complete model includes several parallel pathways originating after the initial visual analysis, each dedicated to the processing of other types of facial information (not considered further in this paper). Reports on brain damaged subjects document dissociations and in some cases double dissociations of symptoms that are consistent with the distinctions among cognitive operations posited in the original Bruce and Young's model. More recent psychological and neuropsychological evidence has prompted modifications [4] or rebuttals [5] of the model, including a substantial revision [6], but the original version has guided ERP research on face recognition over recent decades. It is important to note that all models assume that many different types of memory codes (pictorial, face structural, emotional, social, semantic, episodic, verbal, etc.) are associated with each familiar face, a fact to be remembered when considering the experiments reviewed below.

The notable increase of fMRI studies concerning both face perception and face recognition in recent years has induced the formulation of neurofunctional models which are intended to explain how the distinct functional aspects involved in face processing are supported by brain architecture with components or nodes that are stimulus- and task-dependent, specialized in the processing of different inputs relative to faces [6, 9, 10]. Some neural models try to explain how neural connectivity among certain brain regions (not necessarily close to each other) is required for efficient processing [11, 12]. Haxby et al. [10] proposed that facial information processing is mediated by a hierarchically organized and distributed neural system, composed of both a “core” and an “extended system.” The core system includes three bilateral regions in occipitotemporal visual extrastriate cortex, which are in the inferior occipital gyri (concretely, the region termed Occipital Face Area or “OFA”), in the lateral fusiform gyrus (concretely, the region termed Fusiform Face Area or “FFA”) and in the superior temporal sulcus (concretely, the posterior superior temporal sulcus or “pSTS”). The OFA-FFA link is thought to participate in processing of invariant structural face information (i.e., the identity of the face), whereas the OFA-STS link processes dynamic aspects of faces (such as expression). The extended system comprises limbic areas (for emotion processing) and auditory regions (for paralexical speech perception) among others. These regions, acting in cooperation with the “core” regions, provide pertinent information from other (nonvisual) cognitive domains to enable the processing of face-derived information. In fact, Gobbini and Haxby [9] point out that successful recognition of familiar individuals may also require the participation of the so-called “theory of mind” areas (such as the anterior paracingulate cortex, the posterior superior temporal sulcus (pSTS)/temporoparietal junction (TPJ), and the precuneus), which have been implicated in social and cognitive functions.

According to Ishai [11], the neural connectivity among face sensitive regions depends on the nature of the stimulus and task demands. Thus, seeing faces with affective connotation increases the “effective connectivity” between the fusiform gyrus (FFG) and the amygdala, whereas seeing faces of celebrities or famous persons increases the coupling between the FFG and the orbitofrontal cortex. Additionally, task influence is revealed by the increase in “bottom-up” connectivity between extrastriate visual regions and the prefrontal cortex during face perception, whereas the mental generation of face images increases the “top-down” connectivity (see also [13]). In any event, hitherto, the relationship between processing stages posited in cognitive models and the cortical machinery identified with neuroimaging is not clearly understood, and a direct mapping may not exist.

### 2.2. ERPs Are Essential for the Search of Effective Connectivity between Brain Areas for Face Processing

As outlined above, an extensive catalogue of cortical and subcortical brain areas apparently involved in face processing has been provided by an increasing flow of neuroimaging studies [11]. For some of these areas, unequivocal evidence that they are essential nodes of the brain network involved in face recognition comes from neuropsychological case studies and/or reversible inactivation experiments. For other structures, the evidence is not as clear-cut. In any case, the strength of this approach (mainly involving fMRI and to a lesser degree positron emission tomography, PET) lies in its relatively high spatial resolution and in its precise anatomical localization. However, a present limitation of fMRI (and more so PET) is its poor temporal resolution (in the order of several seconds), which in most studies depends on signals derived from slow hemodynamic responses of the local neural activity of interest. Therefore, although fMRI can target possible nodes of the cortical network of face processing, it offers very limited information on the temporal dynamics of the activation (or inhibition) of those nodes as they participate in face processing.

A complete model of face processing has to specify not only who are the cortical actors but also in what sequence their roles are played out as well as what types of interaction occur among them. Since faces are usually identified in less than half a second, we are dealing with processes that are carried out in a range between ten and several hundred milliseconds. Interestingly, this time range corresponds to the latencies of face-related unit activity recorded in consciously behaving monkeys [14, 15]. Furthermore, despite the excitement generated by studies of functional and “effective connectivity” based on fMRI data (see, e.g., [11]), the time-scale of interactions identified by these studies is necessarily slow due to the nature of the fMRI signals.

This contrast between an increasingly detailed anatomical picture of the nodes comprising the face-processing network on one hand and such meagre knowledge of its temporal dynamics on the other makes it timely to review the ERP research on both face perception and face recognition. ERPs are voltage variations that index the synchronized postsynaptic activity of large neural masses. Although these potentials, measured at the scalp, are difficult to relate to their neural sources, and as a recording technique they have relatively low spatial resolution, they may be recorded with very high temporal resolution. A large body of studies allows us to identify ERP components that are reliably associated to different aspects of face processing. Recently, new promising methods have been developed to infer the neural sources (i.e., the distribution of current sources inside the brain) that generate the scalp-recorded ERPs (see, e.g., [16–18]). These methods have to deal with the difficulties of the “inverse problem” associated with such an inference task, namely, the nonuniqueness of the solution, the limited numbers of sensors available (which makes the problem highly underdetermined), and the instability of the solutions due to the observation noise. However, in conjunction with a now substantial database of intracranial recordings of face-related potentials [19], they provide useful constraints on models of face processing. The ERP technique also has the potential to be integrated with other neuroimaging methods, finding solutions to previously unanswerable questions.

### 2.3. On the Neuronal Origin of ERPs and the Specificity of Neural Mechanisms for Face Processing

Further progress in experimental designs aimed at exploring the brain dynamics of face processing will also be inevitably coupled with the advance in knowledge of the electrophysiological neuronal mechanisms giving place to scalp recorded potentials evoked by face stimuli. A neurobiological reductionist approach, based on the biophysical nature of EEG, has intended to explain both the positive and negative voltage deflections characterizing the ERP waveforms as mirrors at the scalp of the underlying excitatory and inhibitory neuronal activity, occurring in specific cortical layers [20]. Thus, negative ERP components, for example, might be reflecting a massive depolarization of apical dendrites in cortical layer I resulting from thalamocortical excitation as well as an inhibition in deep cortical layers. This neural activity would underlie psychological feed-forward processes like formulation of perceptual “expectancies” and preparatory activation of preexisting cognitive structures. While this approach merits the interest of neuroscientists, in an effort to accommodate within a unifying framework the proliferation of uncountable ERP components, we consider that the incomparable high temporal resolution related to ERP data interpretation offers a unique opportunity to study the complex dynamic nature of cognitive functions such as those involved in face processing.

On the other hand, in line with the most traditional view on ERPs, the effort to characterize specific mechanisms underlying face processing has attracted the attention of research groups in the search for brain responses that, being larger for faces than for other stimuli, can be considered domain-specific [21–24]. Whereas most researchers, on the basis of neuropsychological, developmental, and neuroimaging data, favor the “face specificity” hypothesis, the alternative view sustains that the face superiority effect is a consequence of “expertise”, developed by the greater and earlier experience that we have gained with faces in relation to other visual objects (see [25] for a discussion on this issue). To address the “specificity” question, some authors have carried out some experiments using “objects of expertise” and it is proposed that the kind of processing (i.e., holistic) that characterizes face processing can be the key to understand the functional and neural overlap between face and object processing [26–28]. However, progress in this direction is practically null. It might be sensible to conduct more studies to unveil the functional architecture of the brain system involved in face processing and to show how its components can be investigated using ERPs and other experimental methods [29]. Accordingly, in this work, we focus on studies of ERPs related to visual-structural aspects which reveal that a face is different from other visual objects as well as on those studies concerning the differentiation of individual faces. From a functional point of view, we then refer to experiments on brain responses regarding mainly the structural encoding necessary to activate the “FRUs” and, eventually, the verbal-semantic information associated with each known face (i.e., related to the “core” and “extended” neural systems, resp.).

## 3. Event-Related Potentials as Electrophysiological Markers of Operations Related to Face Processing

### 3.1. Categorization and Initial Structural Processing of Faces Is Revealed in the Early P1 and N170 Waves

Much of the ERP research on faces has searched for face-sensitive responses and has been based on comparing (in both healthy individuals and neurological patients) the brain activity elicited by face presentations with that elicited by the presentation of other categories of visual stimuli (the same comparison that has evidenced the “core” areas in fMRI experiments).

**Figure 1:**
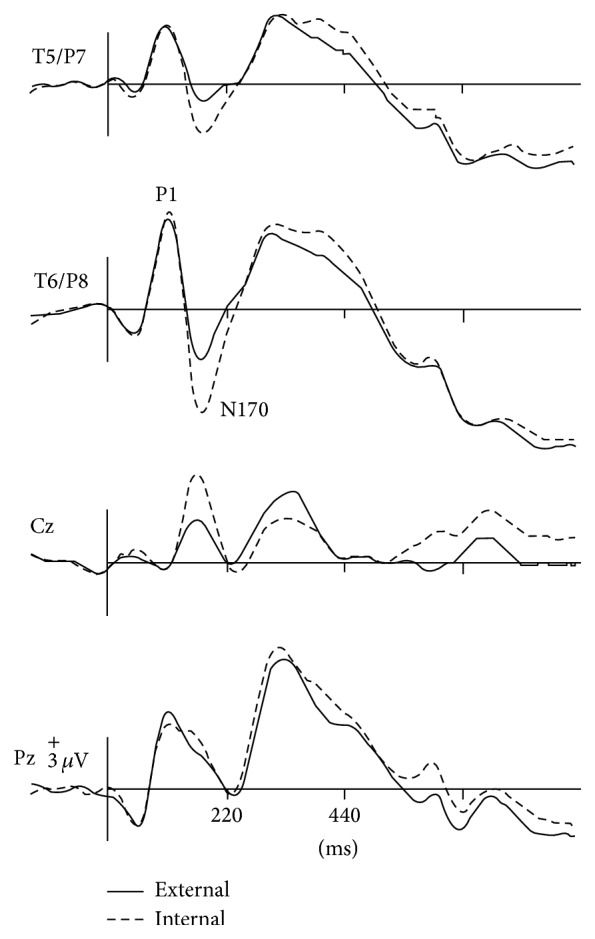
ERPs elicited by external (straight line) versus internal (dotted line) features of familiar faces in a recent experiment [7]. Note that N170 was larger for internal features and enhanced at the right temporal posterior site T6/P8. At the same latency, a positive peak (VPP) was present at the central midline position Cz.

One of the most robust brain responses described in the literature on face processing is the N170 component [30] (Figure 1). N170 is reliably larger for faces than for other categories of visual objects. One notable exception is pictures of front views of cars which elicit a N170 that is comparable with the N170 elicited by upright faces. This is probably due to a relatively invariant face-like feature configuration (see [31, 32]). The second notable exception is pictures of human bodies and body parts, which also cause a conspicuous N170 effect but that is generated in more anterior brain regions, probably concerning body-sensitive cortices [33, 34]. This negative wave has its maximal amplitude at posterior temporal regions (greater on the right side) and neural sources in lateral, basal temporal, and extrastriate occipital cortices have been proposed [30, 35–38]. Many authors additionally suggest that the FFA in the lateral FFG, a region suggested by neuroimaging studies as being especially sensitive to faces [39–41], is involved. However, other authors emphasize a more lateral source in the inferior temporal gyrus or generators in the p-STS [37, 42, 43]. The fact that face-selective N170 could be elicited in a patient with extensive lesions that cover the areas occupied by FFA in normal subjects suggests that N170 has multiple sources [36, 44].

In electrophysiological recordings, with electrodes that were placed subdurally on the cortical surface in neurological (epileptic) patients, a negative potential, N200, was evoked by faces but not by other categories of stimuli [41, 45, 46]. This N200 was located on the left and right fusiform and inferior temporal gyri and can be considered a cortical correlate of the scalp N170. More recently, Barbeau et al. [19], using intracerebral electrodes (placed more profoundly than subdural ones), have also identified a deep neural correlate (although with polarity reversal) of N170. Thus, in old/new tasks regarding face and object recognition, they found a face-sensitive P160 that was recorded in several posterior regions such as the lateral occipitotemporal cortex although mostly in posterior fusiform and lingual gyri.

It was initially suggested that N170 could reflect the activation of a mechanism specialized in initial stages of face structural encoding [30, 47–51]. However, several studies have reported that this wave is sensitive to experimental manipulations linked to subsequent stages of face processing which concern facial contents in LTM. Thus, several authors have found that N170 is modulated by face familiarity or by face repetition within a sequence of visual stimuli ([37, 52–55]; see [56] for a similar result concerning the M170 response described in magnetoencephalography (MEG), but see also [38, 49, 57]), and by the perceptual and contextual experience denoting task-dependent “top-down” processing ([58]; but see also [59]).

Experimental results supporting both alternative explanations still make the interpretation on the functional significance of N170 controversial. However, data provided by deep recordings indicate that the ERP patterns that differentiate familiar and unfamiliar face processing emerge only in those components beyond 200 msec in temporal mesial structures [19], supporting the notion of N170 reflecting a “face detector” mechanism, which triggers the encoding process in the occipitotemporal cortex [21, 47]. Recent evidence for the “face detector” hypothesis has also been offered by neural adaptation experiments. In this case, amplitude reductions of N170 when faces were preceded either by the same face or by different faces were found relative to when they were preceded by other perceptual categories, like objects, voices, or words or when a facial social signal like gaze direction was manipulated [60–64]. Such findings could also explain to some extent certain initial discrepancies among those research groups which obtained a larger N170 when faces were intermixed with other stimulus categories relative to when faces were presented as a unique category in recognition experiments (see, e.g., results from Bentin and Rossion groups and those from Schweinberger and Sommer groups, resp.). Interestingly, amplitude attenuations and latency delays of N170 are usually associated with the removal of internal features [7, 65], but they have also been reported when facial contours are deleted [50]. This suggests that N170 can be associated to a relatively late operation within structural encoding, likely concerning the generation of face gestalts that will contribute further to individual identification.

Around a decade before the initial description of N170 by Bentin et al. [30], other researchers had described an ERP of similar functional characteristics but of inverse (i.e., positive) polarity and maximal amplitude at central sites on the scalp. Bötzel and Grüsser [48] and Seeck and Grüsser [66] observed that the electrophysiological responses to faces differed from those elicited by other serially displayed visual stimuli (a chair, a tree, the human body, different kinds of vases, shoes, tools, and flowers). The principal difference consisted of a positive peak elicited by the face images appearing between 150–190 msec (P150) and a negative peak between 220–300 msec (N300) poststimulus. These face-sensitive responses were more conspicuous at mid-line electrodes (the standard scalp positions from the 10-20 International system Cz, Pz, T5, and T6 were used in those studies), and no lateralization effect was observed. The “vertex positive peak” or “VPP” was the term then proposed by other authors [51, 67, 68] for this brain response also observed when the participants perceived faces presented either as drawings or pictures in different sizes and even as illusory figures resembling faces [69]. Jeffreys [51] and George et al. [69] pointed out that VPP reverses its polarity in the temporal regions and they agree that the location of its neural generators could be those areas in the temporal cortex functionally equivalent to the superior temporal sulcus in nonhuman primates, the inferior temporal cortex, and possibly also (as suggested by [48]) some limbic structures and basal temporal regions.

The critical difference causing researchers to observe alternatively either N170 or VPP was the reference electrode position: whereas those that described the VPP used lateral sites near temporal regions (e.g., mastoid bones or interconnected ear lobules), the posterior temporal N170 was conspicuous when the tip of the nose was used as the reference site in the recording montages (Bötzel et al. [35] and Jeffreys [51] initially alerted of this important methodological issue; see [70] for a study on the importance of reference placement in ERP experiments on face processing). New research using both high-density recordings and appropriate source analysis is necessary to unravel the extent in which both components have overlapping neural generators.

The neural mechanism represented by N170/VPP might be activated subsequently to the perception of certain features suggesting the global form of the perceived object (face), which triggers the process of categorization. In fact, in latencies earlier than 170–200 msec, several studies have also found modulations of both amplitude and latency on positive deflections concerning facial structural processing. Such responses might reflect the encoding of primary sensorial cues necessary for subsequent perceptual integration into more global representations of the facial data. Thus, Linkenkaer-Hansen et al. [71], in a combined ERP-MEG study, proposed that some degree of face-selective processing seems to occur around 100–130 msec, since they observed both amplitude and latency increases of the P1 (P120) to inverted faces (an experimental manipulation that disrupts the holistic processing) but not to upright faces. In the same study, the visual inspection of magnetic field contours and neural source modeling suggested that P1 originated in the posterior extrastriate cortex, whereas N170 was generated more rostrally possibly in the fusiform and inferior temporal gyri. Similar neural sources for P1 and N170 have been reported very recently in another MEG study related to face inversion [72]. Moreover, Halit et al. [73] found that P1 (in the 48–120 msec time window) is larger for atypical faces created artificially by varying the distance among features (Experiments 1 and 2), which denoted, for these authors, the influence of either attentional or “top-down” mechanisms concerning the analysis of a facial prototype. In the same study, the N170 was larger for atypical faces only in Experiment 2 in which the interindividual face typicality processing was evaluated. This was interpreted as an indicator of N170 reflecting the perceptual processing of particular faces in relation to a general facial prototype.

In relation also to the functional role of these early ERPs, in an interesting experiment the spatial frequency of face images varied in order to test the effect of both the coarse and the fine processing on ERPs [74]. In this study, the P1 amplitude was augmented for low-spatial frequency faces, while N170 amplitude was augmented for high-spatial frequency faces. Additionally, the P1 amplitude was unaffected for physically equiluminant faces compared with the response evoked by houses. These results were considered by the authors as evidence of P1 reflecting an early face-sensitive visual mechanism and its holistic process per se which is triggered whenever a stimulus contains sufficient information to generate the concept of face (e.g., gestalt-based). Interestingly, Mitsudo et al. [75] found a larger P1 for upright than for inverted faces when stimuli were presented at a subthreshold duration, which was interpreted as reflecting the activity of a local contrast detector of face parts that can be useful to discriminate faces from objects.

In another study [76], inverted but not upright or contrast reversal faces evoked a delay in P1. Furthermore, in a series of MEG studies, Liu et al. [77] reported that both M100 and M170 (the MEG analogues of P1 and N170, resp.) correlated positively with successful face categorization, whereas only M170 correlated with successful face recognition (see also [78, 79]). Also, M100 was larger for face parts and M170 tended to be more sensitive to facial configuration.

Taking into account the results derived from all these studies, the brain responses P1 and N170 can be considered as relatively early electrophysiological markers of neural mechanisms leading to the formation and activation of face representations. Data on the modulations of both components cited in the preceding paragraphs suggest that the earlier P1 might be an indicator of subroutines responsible for the* grosso modo *detection of any stimulus candidate to be categorized as a face within our visual field. On the other hand, N170 might reflect a subsequent operation of detection of those features contributing to defining a face. It would be facilitated by the presence of a canonical configuration of those stimuli that are potentially facial and that would eventually lead to an adequate identification of exemplars (individuals) at a subordinate level.

### 3.2. Access to Face Representations Is Associated with Activity Beyond 170 msec

Repetition paradigms have been frequently used to ascertain the access to LTM representations [80]. Repeated presentation of the same faces (within relatively short time intervals) induces, compared to nonrepeated stimuli, ERP modulations between 180 and 290 msec poststimulus. Thus, N250r or “ERE” (“early repetition effect”) has been described as a negative ERP peaking at around 250 msec at posterior temporal sites (larger on the right side) with polarity reversal at anterior sites at the same latency [32, 81]. The N250r effect is larger for familiar than for unfamiliar faces. This effect is also larger for nonmasked versus masked stimuli in an explicit matching perceptual task and with respect to face semantic matching tasks. Thus, it does not depend solely on automatic preactivation by face repetition [80], although it can be elicited even in a facial expression detection task where face identities are implicitly activated [82].

The N250r is found even with presentation of different images of the same person, suggesting that it is related to the activation of relatively abstract representations concerning face structure which are invariant over transformations of low-level visual cues [37, 38, 83]. Although N250r does show a degree of image specificity (larger repetition effect across the same image), a study found equivalent priming by the same repeated face image and by the presentation of stretched and unstretched versions of the same face [84], which confirms that N250r does not simply reflect low-level visual (pictorial) coding but is related to person recognition.

On the other hand, N250r has larger amplitude to upright famous faces than to nonhuman primate faces and it is not significant for inverted faces, which links it to face-recognition mechanisms [32]. Moreover, this effect is not obtained with pictures of automobiles in the same experiment or with pictures of hands or houses in a more recent study [33]. In this latter study, the N250r was elicited by the second presentation of faces despite the high perceptual load at initial presentation (see also [85] for a similar result), supporting the notion that a putative face-selective attention module supports encoding under high load and that similar mechanisms are unavailable for other natural or artificial objects. Intriguingly, Henson et al. [86] have reported repetition effects for certain everyday nameable objects in a combined ERP-fMRI experiment. However, contrary to Henson et al., in the studies of Schweinberger et al., faces were presented as task-irrelevant distractors, a crucial difference that might explain such apparently contradictory findings.

In the experiment of Henson et al. [86], a repetition-related positive shift over frontal sites and a transient negative deflection over occipitotemporal sites were produced from 200 to 300 msec only with short repetition lags, supporting the notion that N250r is short-lived [38, 86]. Another repetition effect was found between 400–600 msec by Henson et al. [86], but that was less affected by the increasing lags and it had a central maximum, suggesting that the two effects reflected the activity of at least partially distinct neural generators. A similar distinction between short and long-latency repetition effects for faces was found by Itier and Taylor [76]. All this supports the proposal that N250r indicates the transitory activation of long-term memory representations [63, 64, 82]. Accordingly, Scott et al. [87] found that those modulations occurring around 250 msec could be associated with subordinate-level versus basic-level training, corroborating that in face recognition tasks this ERP is related to processing of representations of individuals.

Source modeling based on high density recordings suggests that the possible neural generators of N250r are located in basal/inferior temporal regions (predominantly on the right side), specifically in the FFG, more rostrally than the estimated generators for N170 [38, 88]. In fact, its possible neuromagnetic correlate, the M250r, also especially sensitive to upright faces versus control stimuli, is predominantly associated with the activity in the right FFG [38]. Accordingly, Henson et al. [86] reported with their fMRI data a decrease in the hemodynamic response (the hemodynamic correlate of stimulus repetition) associated with repetition in several inferior occipitotemporal regions, the magnitude of which also typically decreased as lag increased.

### 3.3. Modulations of Negativities around 400 msec Are Related to the Retrieval of Content from Face Representations and of Its Associated Verbal-Semantic Information

The search for ERP markers concerning face recognition has also motivated researchers to use the rationale underlying experimental tasks originally developed in language studies, which were designed to know the principles of organization in LTM. The N400 component was originally described by Kutas and Hillyard [89], who compared ERPs elicited by the final word of a sentence when it was congruent with the preceding context (“I drink coffee with sugar and milk”) and when it was incongruent (“I drink coffee with sugar and socks”). The N400 was larger for the incongruent ending (which violated contextually generated expectancies) and this component has been used as an index of the degree of contextual preactivation during memory retrieval or of the amount of postretrieval integration with context (see [90] for a review).

By creating different types of contextual expectancy, the retrieval of distinct kinds of memory codes related to faces can be probed with N400-like components [7, 8, 49, 57, 81, 83, 91–102]. Importantly, such responses have different latencies, durations, and topographic distributions depending on the degree of involvement of the verbal information in the task [8, 100].

**Figure 2:**
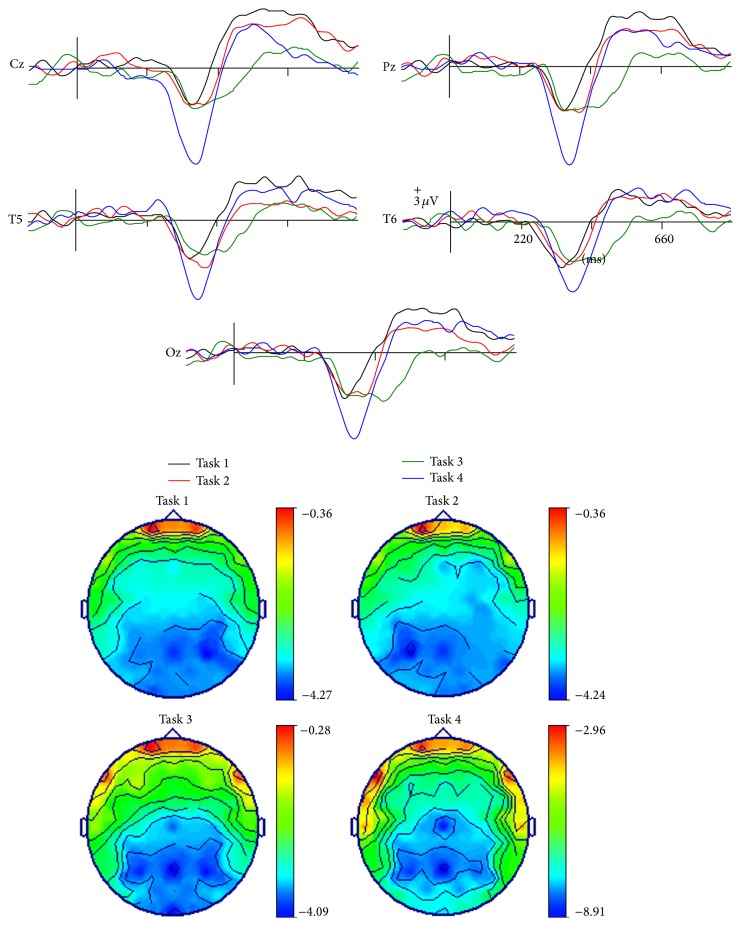
Long-latency ERPs related to face recognition. Top: examples of facial N400-like ERPs (waveforms resulting from subtracting matching trials from mismatching trials) elicited in different tasks in which the degree of verbal and structural visual information involved was varied: a N360 (black) elicited by face-feature mismatching in faces learned without associated verbal information; a N380 (red) elicited by face-feature mismatching in faces learned with occupations and names; a cross-domain N440 (green) elicited by face-occupation mismatching; and a N370 (blue) elicited by occupation-name mismatching. Bottom: topographic voltage maps showing the scalp distribution of these ERPs in each task when the amplitude value was maximal.

The most obvious application of this approach has been to create a context with one face and then to present the same face, a semantically related or unrelated face [91]. In general, the long-latency “incongruence negativities” related to faces that were observed in the above mentioned studies have been elicited by facial stimuli with strongly linked verbal-semantic codes and, in fact, such negativities have been elsewhere associated with domain-independent postperceptual processes [38]. Searching for a more “domain selective” approach, several studies have analyzed face structural processing by presenting incomplete (i.e., removing eyes/eyebrows) familiar faces as primes (i.e., contextual stimuli) and asking participants to detect a feature mismatch in subsequently displayed complete faces. “Incongruent” face-feature completions (putting in place eyes from another face), as compared to congruent completions (correct features), have elicited a negative component around 380 msec which seemed similar to the classical N400 effect [95, 97, 102]. This component is alleged to reflect the lack of associative priming among facial features concerning the face structural representation in LTM. This response has been elicited even by familiar faces for which the names were not known [102], by faces for which the participants possessed only their visual-structural memories since they were artificially learned at the laboratory under a controlled procedure [97, 98, 103], and independently from occupation retrieval [104]. Moreover, a “pure” visual facial N360 has been elicited by structural processing of faces for which verbal-semantic information was not easily available [8] (Figure 2). This N360 was maximal at the right temporal posterior region on the scalp (see compatible result with the N350 from Jemel et al. [95], where neural source estimation was carried out using current dipole localization). Accordingly, N360 might share some neural generators with N170 but is probably representing an ulterior stage in the processing of a known face and tentatively associated with the retrieval from LTM of the visual information stored in the “FRUs” [1].

In summary, the results derived from all these experiments using facial stimuli seem to suggest that N400-like components can be generated in an experimental framework related either to the contextual preactivation for repetition (e.g., in identity-matching tasks, in serial presentation of repeated versus nonrepeated faces) or to association (e.g., in face-feature, face-occupation, or face-pairs matching tasks) related to face memories. However, we want to emphasize that the denomination of such brain responses as electrophysiological markers in the face visual domain should firstly consider the study of the activity elicited by faces independently from other verbal-semantic information which is associated commonly with faces. This verbal-semantic information is, nevertheless, relevant for the eventual identification of those individuals that we know. New experimental studies using high density ERP recordings to improve the spatial resolution of electrophysiological data will allow delineating those possible neural generators of “facial” N400-like waves. Such future studies are necessary to investigate whether face-sensitive neural mechanisms supporting structural processing can be triggered in a relatively independent way from those underlying verbal-semantic processing associated with faces (Table 1).

## 4. New Methods for Further Research

### 4.1. Dynamic Causal Modeling to Disentangle the Dynamics of the Face Processing Network

Most research studies developed up to date, some of which are described here, propose plausible neurofunctional models of different aspects of face processing, based solely on estimates of “where” and “when” the underlying neural events associated with this process occur in the brain. However, the ultimate goal of these models is to describe “how” brain activity is coordinated among different regions during the execution of the given task. For this, several pieces of information critical for characterizing a network are missing. These include the directionality of information transfer or “effective connectivity” between connected regions [105, 106]. In this sense, current developments in both measuring and analysis techniques are providing tools that allow a movement from “guessing” to actually “inferring” neurofunctional network models directly from the data.

In general, “effective connectivity” relies on metrics of interactions that are more or less related to the notion of temporal precedence (because of propagation and synaptic delays) of the activity in the driving structure with respect to that in the driven ones. Due to their high temporal resolution, EEG and MEG are particularly amenable for this type of analysis. In contrast, fMRI is sensitive to changes of local perfusion and oxygen uptake by neurons, which is characterized by the “hemodynamic response function” that delays hemodynamic responses, relative to their hidden neuronal causes. Therefore, fMRI provides an indirect measure of neuronal activity, but the actual nature of this relationship is still a matter of current debate [107]. In addition, the “hemodynamic response function” shows regional variations that make it impossible to estimate neuronal delays directly from the fMRI measurements. This physiological limitation not only compromises the temporal resolution of the technique but also compromises its capability for estimating “effective connectivity” directly from the data [108]. Therefore, despite the exciting knowledge contributed by fMRI and other techniques, ERPs have an important role to play in understanding face processing, but refinement of the analysis techniques is mandatory.

One direction for this development is the use of DCM [109, 110]. DCM relies on a biophysical model that connects the neuronal states to measured responses. It regards an experiment as a designed perturbation of neuronal dynamics in which stimuli cause changes in neuronal activity that are propagated throughout a system of coupled anatomical nodes or sources, which in turn cause changes in the observed EEG/MEG signals. Experimental factors can also change the parameters or causal architecture of the network producing the observations. The inversion of these models is used to infer the “effective connectivity” among unobserved neuronal states and how “effective connectivity” depends upon either stimulus attributes or experimental context. Additionally, Bayesian inference allows the comparison of a set of models with different directed connections and the identification of the optimal model given the data.

As a relevant example for the present work, David et al. [111] carried out a DCM analysis of ERPs recorded during the perception of both faces and houses. As a result, category-selectivity, as indexed by the face-selective N170, could be explained by category-specific differences in forward connections from sensory to higher areas in the ventral stream. Specifically, there was an increase of forward connectivity in the medial ventral pathway from retrosplenial cortex to parahippocampal place area when processing houses* versus* faces. Conversely, in agreement with Haxby et al.'s [10] model, there was an increase in coupling from inferior occipital gyrus (IOG) to the FFA and from IOG to the STS during face perception. The face-selectivity of STS responses was smaller than in the FFA due to a gain in sensitivity to inputs from IOG. The connections from V1 to IOG showed no selectivity. This suggests that category-selectivity emerges downstream from IOG, at a fairly high level, somewhat contrary to expected [112]. In a related study, Fairhall and Ishai [113] used DCM on fMRI data while subjects processed emotional and famous faces. In accordance with David et al. [111], they predicted a ventral rather than dorsal connection between the “core” (visual areas) and the “extended” (limbic and prefrontal regions) systems during face viewing. They also found that the core system is hierarchically organized in a predominantly feed-forward fashion, with the IOG exerting influence on the FFG and on the STS. Furthermore, the FFG was found to exert a strong causal influence on the orbitofrontal cortex (OFC) when processing famous faces and on the amygdala and inferior frontal gyrus when processing emotional faces.

In a recent and pioneering study, Nguyen et al. [114] used DCM as a data fusion approach to integrate concurrently acquired EEG and fMRI data to examine the association between the N170 of ERPs and the activity within the face-selective fMRI network for processing both upright and inverted faces. Data features derived from EEG were used as contextual modulators on fMRI-derived estimates of effective connectivity between key regions of the face perception network. As main results they obtained that the OFA acts a central “gatekeeper,” directing visual information to the STS and the FFA and to a medial region of the fusiform gyrus (mFG). The connection from the OFA to the STS was strengthened on trials in which N170 amplitudes to upright faces were large. In contrast, the connection from the OFA to the mFG, an area known to be involved in object processing, was enhanced for inverted faces particularly on trials in which N170 amplitudes were small. According to these authors, their approach can be considered asymmetric within the model-driven data fusion framework; that is, the forward model (from sources to observable data) is confined here to only one modality (neurovascular coupling from neural states to BOLD signal), whereas the second modality (EEG) is used to constrain that model. In turn, a symmetric approach would rely on a joint forward model that generates both EEG and fMRI data from the same neuronal states. This would allow an integrative model inversion that could take advantage of the exquisite temporal resolution of EEG data and greater spatial resolution of the BOLD signal [115].

Data fusion approaches are a direct consequence of recent hardware and software developments, which have made it feasible to acquire EEG and fMRI data simultaneously. Nonetheless, this approach should be applied cautiously since the degree of overlap between underlying neuronal activity generating observations in each modality is variable and, for the most part, unknown [116]. Specifically, some studies related to face processing have shown that different ERP deflections correlate best with the BOLD (blood oxygen level dependent) response; for example, P3a is related to BOLD signal changes in the right fusiform and left superior temporal gyrus for a facial emotion recognition task [117] and N170s for face and house visual stimuli have been found to correlate well with hemodynamic responses in various brain areas in the temporal-occipital lobes [118]. These findings imply that certain EEG components may correlate better with the BOLD signal than others. Moreover, the relationship between these components and the BOLD response may vary according to the experimental paradigm used. Thus, although EEG-fMRI fusion has great potential to pursue new strategies in cognitive neuroimaging, including those with respect to face processing, further studies about the actual nature of the coupling between the underlying neuronal activity and these two types of measurements are necessary. This will allow the formulation of more realistic forward generative models as well as the development of appropriate multimodal inference methods.

### 4.2. Single-Trial Perspective in the Study of Face-Sensitive ERPs

Another direction for future development is that relative to single-trial analyses of evoked activity. In the last years an increasing number of studies are aimed to explore EEG processes whose dynamic characteristics are also correlated with behavioral changes but cannot be seen in the averaged ERP [119, 120]. Comparing different procedures for single-trial data filtering (viz., raw sensor amplitudes, regression-based estimation, bandpass filtering, and independent component analysis or ICA), De Vos et al. [121] found the best single-trial estimation for ICA in the case of the N170 single-trial ERP. According to such findings the face-sensitive N170 does not represent activity from a face-tuned neuronal population exclusively but rather the activity of a network involved in general visual processing. Moreover, single-trials approach has allowed Rousselet et al. [122] to know that the “N170 face effect” is essentially characterized by an event-related modulation of amplitude from trial to trial rather than an increase in phase coherence in the N170 time window.

The single-trial approach combined with parametrically manipulated stimuli is intended to establish statistical links between image properties and brain activity [123–125]. Implementations of such analyses are based on the criterion that information content of brain states can only be revealed using reverse correlation techniques and statistical modeling approaches by determining what global and local image properties modulate single-trial ERPs [125].

The single-trial perspective has also shed light on the nature of the neurocognitive deficit in prosopagnosic individuals. In a recent study, Nemeth et al. [126] found that the altered (reduced) face sensitivity of the N170 in congenital prosopagnosia was due to a larger than normal N170 to noise stimuli rather than to a smaller N170 elicited by faces. This effect was explained, on a single-trial basis, by a larger oscillatory power and phase-locking in the theta frequency-band around 130–190 ms as well as by a lower intertrial jitter of the response latency for the noise stimuli.

### 4.3. Face Processing and Brain Oscillations

**Figure 3:**
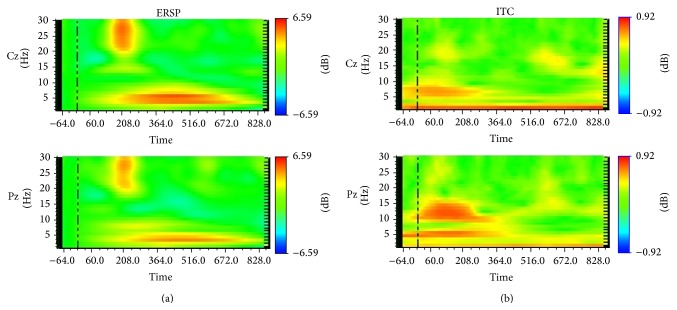
Time-frequency plots derived from wavelet transformations of multiple EEG trials in a subject. Induced activity in form of event-related spectral perturbation (ERSP (a)) and of the inter-trial phase coherence (ITC) as a measure of phase consistence among trials (b), both represented for recording sites Cz and Pz of the International 10/20 System and elicited in a face-feature matching task (see, e.g., [8]). Observe how induced activity (ERSP) is larger (red colour) in the middle of the epoch for low frequencies and around 200 msec for high ones. In turn, ITC is larger for very low frequencies along the epoch and for other somewhat higher oscillations at the beginning of the epoch.

The development of computing and methodological tools for signal processing in laboratories devoted to electroencephalographic (EEG) research has increased notably in the last decades the interest for the study of brain oscillations and allowed the advance in the interpretation of their functional meaning [119]. A consequence of this development is that evoked responses are no longer considered mere increases in signal amplitude with fixed time course and fixed polarity, arising overlaid on “spontaneous EEG” and detected via trial averaging. Instead, they are thought to reflect, at least partially, a reset of ongoing oscillations and are mainly studied via time-frequency analyses ([120, 127–132]) (Figure 3).

Whereas the assumption of either the (traditional) evoked model or the oscillatory model to understand the event-related EEG activity is controversial, integrative approaches that analyze simultaneously both types of scalp-recorded data are necessary to elucidate the brain mechanisms underlying cognitive processes of interest (see, e.g., [130], and their proposal of the “event-related phase reorganization” model).

In relation with face processing, the face sensitive scalp-recorded N170 has been related to modulations of amplitude of low frequency (in the 5–15 Hz band) oscillations [122]. In fact, Tang et al. [133] have differentiated this low-frequency (4–10 Hz in their study) oscillatory activity from a lower (0–5 Hz) frequency accounting for the (usually considered positive counterpart of N170) vertex positive peak (VPP), suggesting that both ERPs have different sources.

On the other hand, Anaki et al. [134] studied the N170 wave conjointly with induced gamma band activity (>20 Hz), while face orientation and face familiarity were manipulated. These authors found that N170 was modulated by inversion but not by familiarity, whereas low (25–50 Hz) and high (50–70 Hz) gamma(s) were modulated by orientation and familiarity, respectively. In a similar vein, Zion-Golumbic and Bentin [65] dissociated the functional roles of N170 and induced gamma oscillations when they found that, unlike the N170, the amplitude of gamma was sensitive to the configuration of internal facial features but insensitive to their presence within or outside a face contour. A relatively late gamma sensitivity and an increased P2 concerning the own-race effect were both reported by Chen et al. [135] who in turn did not find any race modulation on the “structural” N170 component. These authors suggested that such modulations could be associated with more elaborated processing on the basis of configural computation due to greater experience with own-race faces. Furthermore, using subdural recordings in the ventral occipitotemporal cortices, Engell and McCarthy [136] found that both N200 and induced gamma activity had stimulus (face) specificity; however, only N200 was evoked by impoverished face stimuli that did not induce gamma activity. It suggested that the face-induced gamma response reflects elaborative processing of faces, while face-N200 may reflect a synchronizing event within the face network. All these results suggest that, even in the same latencies, ERPs and neural oscillations can be reflecting distinct neural subroutines and might arise from the activity of separated neural assemblies acting conjointly to make face recognition efficient.

## 5. Conclusions

The study of ERPs concerning face processing has allowed the identification of possible markers for distinct cognitive operations involved in face perception and face recognition. Both the latencies and the scalp distribution of these brain responses as well as the experimental variables modulating their amplitudes allow us to characterize these noninvasively recorded signals as electrophysiological correlates of distinct modules commonly described in the theoretical models of face processing. Thus, the initial processing of faces as complex visual stimuli can be indexed by the early occipital P1, which might be linked to the detection of certain primary structural aspects (for instance, a contour) suggesting the presence of stimuli resembling faces. N170 seems to be more clearly sensitive to detection of faces as complex organized visual stimuli and to the presence of its defining features, prior to intracategorical identification, whereas the later N250r and N400 could be indexes of processes of access and retrieval of information corresponding to long-term face representations, respectively. All these responses can originate in activity of neural populations situated mainly in cortical regions encompassing the so-called “ventral visual stream,” which is assumed to be hierarchically organized from the extrastriate early visual cortices to the temporal regions in accordance with the latencies of such responses.

The high temporal resolution of the ERPs study offers an ideal framework to incorporate new methodological approaches, such as time-frequency and single-trial analyses, to determine, for example, how certain image properties are linked to brain activity. DCM can also benefit from this in order to infer information flow through the face network and the effective connectivity among brain regions, depending on the nature of faces and the task at hand.

The use of methodological tools and perspectives as those mentioned above, together with the enormous and increasing volume of experimental data, can lead to a major breakthrough in the study of the neural dynamics of cognitive operations such as those involved in face processing.

## Figures and Tables

**Table 1:** Summary of the main characteristics of different event-related potentials (ERPs) related to face processing.

ERP (Latency in msec) *Functional significance *	Modulated by	Topography	Possible generators in
P1 (100–130) *Face detector*, *primary cues *	(i) Face parts (ii) Inversion (iii) Typicality (iv) Low-spatial frequency (v) Stimulus duration (threshold for detection) (vi) Categorization	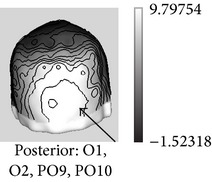	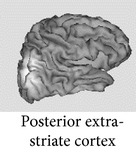

N170 (150–200) *Face detector gestalt-based*, *structural encoding *	(i) Faces versus objects (including within-category adaptation) (ii) Face structure: configuration, inversion, and missing features (iii) Interindividual typicality (iv) High-spatial frequency (v) Familiarity? (controversial)	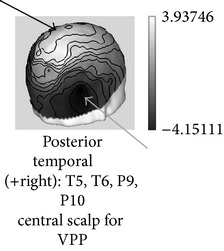	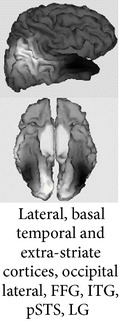

N250r (200–300) *Access to face recognition units in LTM *	(i) Faces versus objects (ii) Repetition (iii) ISI duration (iv) Perceptual masking (v) Present across different pictures (vi) Familiarity	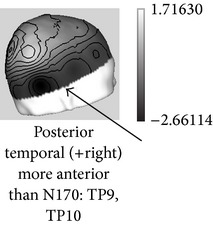	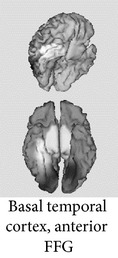

N400-like components (300–500) *Retrieval of face memories including verbal/semantic *	(i) Face structural congruence in familiar faces (face-feature matching) (ii) Associated person information	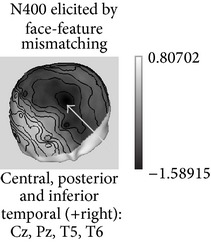	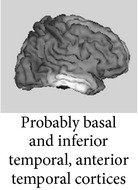

FFG: fusiform gyrus; ISI: Interstimulus interval; ITG: inferior temporal gyrus; LG: lingual gyrus; LTM: long-term memory; pSTS: posterior superior temporal sulcus; VPP: vertex positive peak. The possible locations for ERP generators (last column) are depicted in lighter grays.
